# 

**Published:** 1901-07-15

**Authors:** 


### ANNOUNCEMENTS.

The National Dental Society will meet at Milwau kee Tuesday, August 6th, for a four days' session.

The National Association of Dental Faculties will meet at Milwaukee Thursday, August 1st, for a two days' session.

The National Association of Dental Examiners will meet at Milwaukee Friday, August 2d, for a two days' session.

